# High PTPN13 expression in high grade serous ovarian carcinoma is associated with a better patient outcome

**DOI:** 10.18632/oncotarget.21175

**Published:** 2017-09-21

**Authors:** Véronique D’Hondt, Magalie Lacroix-Triki, Marta Jarlier, Florence Boissiere-Michot, Carole Puech, Peter Coopman, Dionyssios Katsaros, Gilles Freiss

**Affiliations:** ^1^ IRCM, Institut de Recherche en Cancérologie de Montpellier, Montpellier F-34298, France; ^2^ Institut régional du Cancer de Montpellier, Montpellier F-34298, France; ^3^ Département de Biologie et Pathologie Médicales, Gustave-Roussy Cancer Campus, 94805 Villejuif cedex, France; ^4^ Unité de Biométrie, Institut régional du Cancer de Montpellier, Montpellier F-34298, France; ^5^ Unité de Recherche Translationnelle, Institut Régional du Cancer de Montpellier, Montpellier F-34298, France; ^6^ INSERM, U 1194, Montpellier F-34298, France; ^7^ Université de Montpellier, Montpellier F-34090, France; ^8^ Azienda Ospedaliero-Universitaria Cittadella Salute, Presidio S. Anna and Department of Surgical Science, Gynecology, University of Torino, Torino, Italy

**Keywords:** high-grade serous ovarian carcinoma, PTPN13, 4q LOH, tyrosine phosphatase, prognosis

## Abstract

**Background:**

Chromosome 4q loss of heterozygosity (LOH) is frequently observed in high-grade serous ovarian carcinoma (HGSOC). However, this LOH has not been clearly associated with the inactivation of any tumor suppressor gene(s). As the tumor suppressor gene *PTPN13* is located on chromosome 4q21, we investigated its expression in HGSOC.

**Methods:**

PTPN13 protein expression was investigated by immunohistochemistry (IHC) in normal ovary epithelium and in 30 HGSOC samples, whereas *PTPN13* mRNA expression was quantified by RT-PCR in another independent cohort of 28 HGSOC samples. Patients in both cohorts were followed for more than 8.5 years.

**Results:**

PTPN13 protein expression was lower in one third of HGSOC samples compared with normal ovary epithelium. In both cohorts, high PTPN13 expression level (mRNA or protein) in the tumor was associated with favorable outcome and significantly longer survival (HR=0.27; p=0.0087 and HR=0.42; p=0.03, respectively).

**Conclusion:**

This study demonstrates, for the first time, that high PTPN13 expression level is a prognostic indicator of favorable outcome in patients with HGSOC. This finding, in conjunction with our previous mechanistic studies, suggests that PTPN13 loss, possibly by 4q LOH, enhances HGSOC aggressiveness and highlight the interest of studying PTPN13 signaling in HGSOC to identify new potential therapeutic targets.

## INTRODUCTION

Epithelial ovarian carcinoma (EOC) is the first cause of death from gynecological cancers in Western countries. EOC is a heterogeneous group that includes several tumor subtypes with different genetic risk, pathophysiology, clinical behavior, response to treatment, and prognosis. High grade serous ovarian carcinoma (HGSOC) represents 60 to 70% of all EOCs and accounts for the majority of deaths caused by EOC [1]. The Cancer Genome Atlas (TCGA) project has investigated its molecular features in a collection of 316 HGSOC samples and identified mutations of *TP53* in 96% of specimens, of *BRCA 1* or *2* in 22% and of seven other genes in 2 to 6% of samples [2]. It also found that homologous recombination is defective in almost half of the analyzed tumors, and that NOTCH, FOXM, and PI3K signaling are involved in HGSOC pathophysiology [2]. The identification of four molecular subtypes of HGSOC by gene expression profiling (mesenchymal, immunoreactive, differentiated and proliferative) with distinct molecular characteristics and prognosis [3, 4] also illustrates the diversity and complexity of this EOC subtype and justifies the search of prognostic markers to guide therapeutic decision-making.

Chromosome 4q loss of heterozygosity (LOH) has been observed in 39% of EOC, particularly in 67% of HGSOC [2, 5]. Differently from 17q LOH the effects of which have been largely attributed to the tumor suppressor *TP53* located at 17p13, inactivation of potential tumor suppressor gene(s) associated with 4q LOH has not been clearly reported. The *PTPN13* gene is located on chromosome 4q21 [6] and encodes the protein tyrosine-phosphatase non-receptor type 13 (PTPN13, also called FAP-1, PTP-BAS, PTP1E), the non-receptor type protein tyrosine phosphatase (PTP) with the highest molecular weight (270 kDa) [7, 8]. Little is known about its physiological role. Mice lacking the tyrosine phosphatase activity of PTP-BL (the PTPN13 mouse homolog) show mild impairment of motor nerve repair [9]. Our group described PTP-BL role in adipocyte differentiation [10], and also reported the first evidence of PTPN13 negative regulation of tumor growth through an anti-growth factor effect in human breast cancer cell lines incubated with anti-estrogens [11–13]. Other groups confirmed *PTPN13* tumor suppressor gene properties [14–17] and its role in tumor sensitivity to tyrosine kinase inhibitors [18]. Its expression is frequently down-regulated or silenced through promoter hyper-methylation or LOH in several tumor types [19–21]. Moreover, in a large study on colorectal carcinoma, *PTPN13* was identified as one of the three most frequently mutated PTPs and some of these *PTPN13* mutations were also found in tumors from other tissues [22]. However, other studies suggest that PTPN13 acts as a tumor promoter via inhibition of Fas-induced apoptosis [23], or by undefined mechanisms in Ewing's sarcoma [24].

In breast cancer cell lines, we showed [25] that PTPN13 has a negative effect, via insulin receptor substrate 1 (IRS1) dephosphorylation, on the activation of PI3K, one of the driver pathways in ovarian cancer [2]. However, Reed's group found an association between PTPN13 expression and resistance to Fas-induced apoptosis in ovarian cancer cells lines [26], which makes difficult to predict the role of PTPN13 in each tumor type [8, 27]. In addition, *PTPN13* mRNA expression is an independent prognostic marker of increased overall survival in breast cancer [28], in hepatocellular carcinoma [29], and lung cancer [17].

Based on these results and *PTPN13* chromosomal location, we decided to study the expression of PTPN13 in HGSOC samples by reverse transcription and real time polymerase chain reaction (RT-PCR) in a first cohort of 28 HGSOC specimens and then by immunohistochemistry (IHC) in a second independent cohort of 30 HGSOC samples.

## RESULTS

### *PTPN13* mRNA expression in HGSOC

RT-PCR analysis of *PTPN13* mRNA expression in 28 HGSOC samples (Table 1A) showed that *PTPN13* was expressed in all tumors. Moreover, the median expression value in tumors, relative to SKOV3 cells (used as an internal standard for *PTPN13* mRNA expression), was 1.55 (range, 0.066 to 14.03). In 16 HGSOC samples, *PTPN13* expression was higher than in SKOV3 cells, while it was very low (at least 8-fold lower than the median value) in 6 tumors. *PTPN13* expression was not affected by the patients’ age, whereas it was significantly lower in cancers with high International Federation of Gynecology and Obstetrics (FIGO) tumor stage (stage 1-2 vs 3-4; p=0.05) and with suboptimal cytoreductive surgery (optimal vs suboptimal cytoreductive surgery; p=0.007) (Table 2A) (Supplementary Figure 1). As expected, the FIGO stage was correlated with cytoreductive surgery completeness (p<0.01).

**Table 1:** Characteristics of the two cohorts of patients with HGSOC

A: RT/PCR cohort	
**Median age at diagnosis (range), years**	58.5 (25-71)
***PTPN13*** **median expression (range)**	1.55 (0.066-14.03)
**FIGO stage n (%)**	
1-2	6 (22.2)
3-4	21 (77.78)
**Cytoreductive surgery n (%)**	
Optimal	11 (39.29)
Suboptimal	17 (60.71)
**Recurrence n (%)**	
NO	8 (28.75)
YES	20 (71.43)
**Death n (%)**	
NO	9 (32.14)
YES	19 (67.86)
**B: IHC cohort**	
**Median age at diagnosis (range), years**	59 (34-85)
**PTPN13 n (%)**	
IRS	14 (46.67)
IRS ≥median (IRS=8)	16 (53.33)
**FIGO stage n (%)**	
1-2	5 (16.67)
3-4	25 (83.33)
**Recurrence n (%)**	
NO	6 (20)
YES	24 (80)
**Death n (%)**	
NO	4 (13.33)
YES	26 (86.67)

**Table 2A:** Correlation between PTPN13 mRNA expression level and clinical parameters (RT-PCR analysis)

Parameter	PTPN13				Total	%	p-value
	≤ 1.55		> 1.55				
	n	%	n	%			
**Age**							0.131
≤58 years	5	35.71	9	64.29	14	50.00	
>58 years	9	64.29	5	35.71	14	50.00	
**FIGO stage**							**0.050**
1 – 2	1	7.14	5	38.46	6	22.22	
3 – 4	13	92.86	8	61.54	21	77.78	
**Cytoreductive surgery**							**0.007**
OCS	2	14.29	9	64.29	11	39.29	
SCS	12	85.71	5	35.71	17	60.71	

Patients (n=28) were stratified according to the tumor *PTPN13* mRNA expression (≤ or > than the median value of 1.55). PFS: progression-free survival; OS: overall survival; HR: hazard ratios; OCS: optimal cytoreductive surgery; SCS: suboptimal cytoreductive surgery.

**Table 2B:** Assessment of prognostic factors by Cox univariate (RT-PCR analysis)

PFS				
Parameter	n	Events	HR, 95% CI,(Cox model)	p-value(Log rank test)
**PTPN13**				**0.0101**
PTPN13 ≤1.55	14	12	1	
PTPN13 >1.55	14	8	0.32 (0.13 - 0.79)	
**Age**				0.8790
≤58 years	14	10	1	
>58 years	14	10	1.07 (0.44 - 2.58)	
**FIGO stage**				**0.0014**
1 – 2	6	1	1	
3 - 4	21	19	13.51 (1.77 - 103.04)	
**Cytoreductive surgery**				**<0.0001**
OCS	11	3	1	
SCS	17	17	37.40 (4.74 - 295.45)	
**OS**				
**Factor**	**n**	**Events**	**HR, 95% CI(Cox model)**	**p-value(Log rank test)**
**PTPN13**				**0.0087**
PTPN13 ≤1.55	14	12	1	
PTPN13 >1.55	14	7	0.27 (0.092 – 0.77)	
**Age**				0.7740
≤58 years	14	9	1	
>58 years	14	10	1.14 (0.46 - 2.82)	
**FIGO stage**				**0.0006**
1 – 2	6	0	1	
3 - 4	21	19	1.08e+16 (0 – …)	
**Cytoreductive surgery**				**<0.0001**
OCS	11	2	1	
SCS	17	17	3.24e+16 (0 –...)	

Patients (n=28) were stratified according to the tumor *PTPN13* mRNA expression (≤ or > than the median value of 1.55). PFS: progression-free survival; OS: overall survival; HR: hazard ratios; OCS: optimal cytoreductive surgery; SCS: suboptimal cytoreductive surgery

In the 28 patients from whom these HGSOC samples were obtained, the median progression-free survival (PFS) time was 2 years (95% confidence interval, CI, [1.18 - 4.46]) and the 5-year PFS rate was 32.1% (95% CI [16.2% - 49.3%]). Then, patients were divided in two groups according to the median tumor *PTPN13* mRNA expression level (≤1.55: low PTPN13 group; >1.55: high PTPN13 group). The median PFS time was 1.2 years (95% CI [0.44 - 1.91]) in the low PTPN13 group, and 4.5 years (95% CI [1.96 - not reached]) in the high PTPN13 group. Similarly, the 5-year PFS rate was 14.3% (95% CI [2.3% - 36.6%]) in the low PTPN13 group and 50.0% (95% CI [22.9% - 72.2%]) in the high PTPN13 group.

In the whole population, the median overall survival (OS) time was 3.6 years (95% CI [2.72 – 9.90]) and the 5-year OS rate was 46.4% (95% CI [27.6% - 63.3%]). The median OS time was 3.2 years (95% CI [1.36 – 4.75]) in the low PTPN13 group and 9.2 years (95% CI [2.72 – not reached]) in the high PTPN13 group. The 5-year OS rate was 21.4% (95% CI [5.2% - 44.8%]) in the low PTPN13 group and 71.4% (95% CI [40.6% - 88.2%]) in the high PTPN13 group.

**Figure 1:**
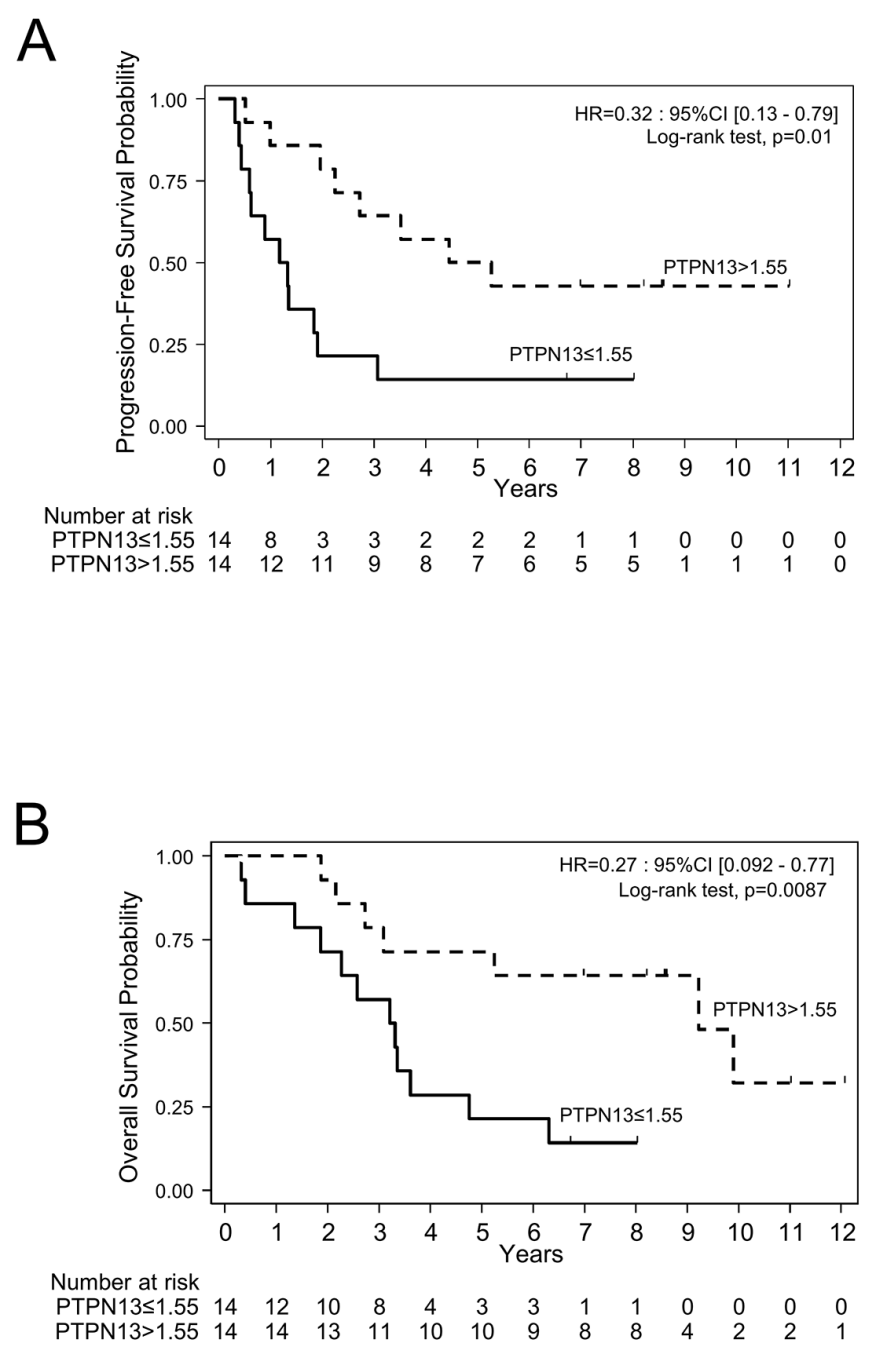
Progression-free survival **(A)** and overall survival **(B)** in a cohort of patients (n=28) grouped according to *PTPN13* mRNA expression levels (≤ or > than the median expression level) assessed by RT/PCR.

Univariate Cox regression analysis showed that PFS and OS were significantly longer in patients with high tumor *PTPN13* mRNA expression (HR=0.32, p=0.0101; and HR=0.27, p=0.0087, respectively) (Figure 1; Table 2B). As expected, classical prognostic factors for OS and PFS (tumor stage and cytoreductive surgery) also were significantly correlated with PFS and OS (Table 2B). However, due to the small sample size and the bad prognosis of this population, there was an imbalance in the distribution of the number of events between categories for these factors. Therefore, the hazard ratios (HR) and 95% CI upper limits were overestimated or not reached. For this reason, multivariate analysis including cytoreductive surgery completeness and FIGO stage was not considered for this dataset.

### PTPN13 protein expression in HGSOC

**Figure 2:**
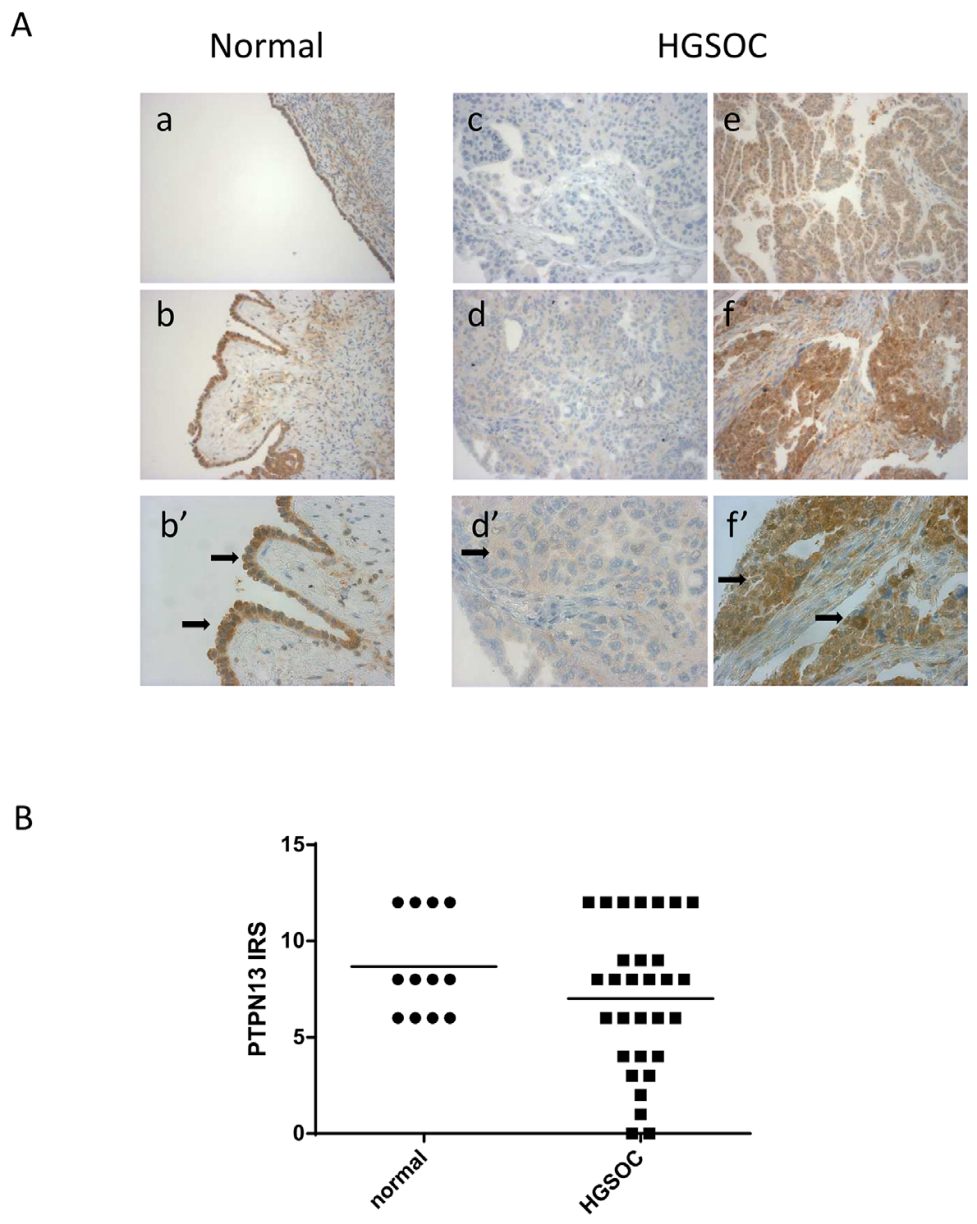
PTPN13 protein expression in normal ovary and HGSOC samples (different from those used for RT-PCR) **(A)** Examples of PTPN13 staining intensity in normal ovary (a: moderate; b and b’: strong) and HGSOC tissue sections (c: negative; d and d’: weak; e: moderate; f and f’: strong). At higher magnification (b’, d’, f’), note the positive signal in epithelial (b’) and malignant epithelial cells (d’, f’) (arrows). **(B)** PTPN13 expression quantification in 12 normal ovary and 30 HGSOC samples. IRS: immunoreactive score calculated by multiplying the percentage of positive epithelial cells (<1%=0; 1-30%=1; 31-50%=2; 51–80%=3; >80%=4) by the staining intensity (negative=0; weak=1; moderate=2; strong=3).

PTPN13 protein expression was assessed by IHC in a second independent cohort of 30 HGSOC samples (Table 1B) and in 12 normal ovary samples. The mean immunoreactive scores (IRS) were 8.7 for normal ovaries and 7 for HGSOC (not statistically different). PTPN13 protein expression was homogeneous in the normal ovary samples with 70 to 100% of positive epithelial cells (moderate to strong staining). Conversely, it was heterogeneous among the 30 HGSOC samples with two negative tumors and seven poorly reactive samples (Figure 2). No correlation was found between PTPN13 expression and the available clinical parameters (FIGO stage and age) (Table 3A).

**Table 3A:** Correlation between PTPN13 protein expression and clinical parameters (IHC analysis)

Parameter	PTPN13 IRS				Total	%	p-value
	< 8		≥ 8				
	n	%	n	%			
**Age**							0.143
≤59 years	5	35.71	10	62.50	15	50.00	
>59 years	9	64.29	6	37.50	15	50.00	
**FIGO stage**							0.743
1 – 2	2	14.29	3	18.75	5	16.67	
3 – 4	12	85.71	13	81.25	25	83.33	

Patients (n=30) were stratified according to *PTPN13* expression (< or ≥ than the median IRS = 8). PFS: progression-free survival; OS: overall survival; HR: hazard ratios; IRS: immunoreactive score; OCS: optimal cytoreductive surgery; SCS: suboptimal cytoreductive surgery.

**Table 3B:** Assessment of prognostic factors by Cox univariate and multivariate analyses (IHC analysis)

PFS univariate				
Parameter	n	Events	HR, 95% CI(Cox model)	p-value(Log rank test)
**PTPN13**				0.2040
**PTPN13 <8**	14	13	1	
**PTPN13 ≥8**	16	11	0.59 (0.26 – 1.34)	
**Age**				0.4409
≤59 years	15	11	1	
>59 years	15	13	1.37 (0.61 – 3.07)	
**FIGO stage**				**0.0042**
1 – 2	5	1	1	
3 - 4	25	23	10.83 (1.44 – 81.28)	
**OS univariate**				
**Parameter**	**n**	**Events**	**HR, 95% CI(Cox model)**	**p-value(Log rank test)**
**PTPN13**				**0.0303**
PTPN13 <8	14	14	1	
**PTPN13 ≥8**	16	12	0.42 (0.19 – 0.94)	
**Age**				0.0955
≤59 years	15	12	1	
> 59 years	15	14	1.93 (0.89 – 4.24)	
**FIGO stage**				0.0704
1 – 2	5	3		
3 - 4	25	23	2.92 (0.87 – 9.81)	
**OS multivariate**				
**Parameter**			**HR, 95% CI(Cox model)**	**p-value**
**PTPN13**				0.058
PTPN13 **<8**			1	
PTPN13 ≥**8**			0.46 (0.20 - 1.03)	
**FIGO stage**				0.123
1 – 2			1	
3 - 4			2.60 (0.77 – 8.77)	

Patients (n=30) were stratified according to *PTPN13* expression (< or ≥ than the median IRS = 8). PFS: progression-free survival; OS: overall survival; HR: hazard ratios; IRS: immunoreactive score; OCS: optimal cytoreductive surgery; SCS: suboptimal cytoreductive surgery.

The median PFS time was 2.2 years (95% CI [1.06 – 2.85]) for these 30 patients with HGSOC and the 5-year PFS rate was 20% (95% CI [8.1% – 35.6%]). The median PFS time was 1.3 years (95% CI [0.87 – 2.85]) in the low PTPN13 group (tumor PTPN13 expression lower than the median IRS which is 8), and 2.7 years (95% CI [0.85 – not reached]) in the high PTPN13 group (tumor PTPN13 expression ≥ 8). The 5-year PFS rate was 7.1% (95% CI [0.45% – 27.5%]) in the low PTPN13 group and 31.3% (95% CI [11.4% – 53.7%]) in the high PTPN13 group.

Univariate Cox regression analysis showed that the PFS differences between groups were not statistically significant. Only a trend was observed for a better outcome in the high PTPN13 subgroup (HR=0.59; p = 0.2). This lack of significance might be due to the small sample size. In univariate and multivariate analyses, only the FIGO stage was a prognostic factor for PFS (Table 3B).

The median OS time for the whole population was 4.1 years (95% CI [2.3 – 5.4]) and the 5-year OS rate was 40% (95% CI [22.8%; 56.7%]). The OS time was 3.2 years (95% CI [1.48 – 4.60]) in the low PTPN13 group and 5.3 years (95% CI [2.30 – 9.83) in the high PTPN13 group. The 5-year OS rate was 21.4% (95% CI [5.2% – 44.8%]) in the low PTPN13 group and 56.3% (95% CI [29.5% – 76.2%]) in the high PTPN13 subgroup.

**Figure 3:**
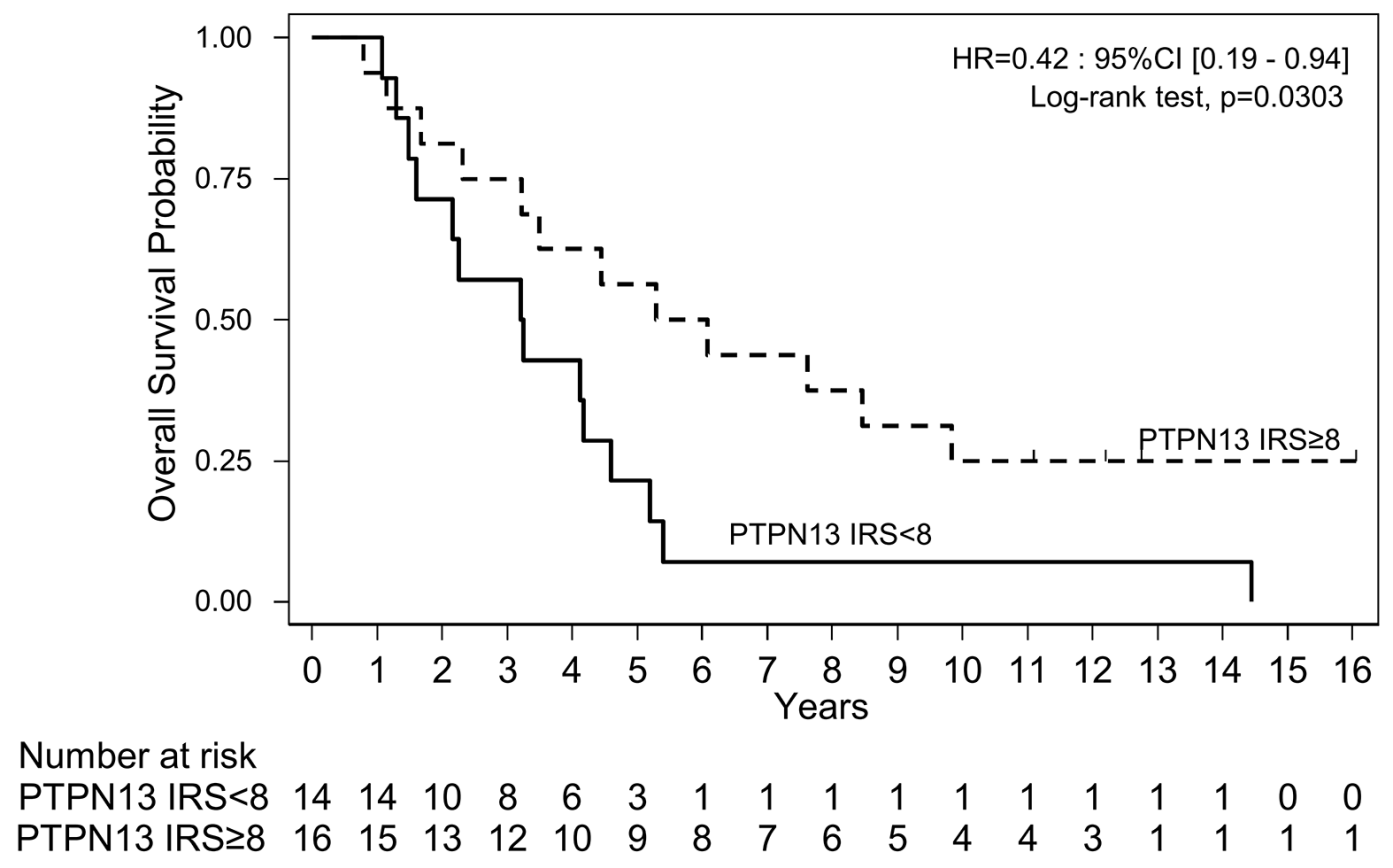
Overall survival in the cohort of patients with HGSOC samples (n=30) grouped according to the tumor PTPN13 expression level (≥ or < than the median IRS=8) assessed by immunohistochemistry

Univariate Cox regression analysis showed that the OS differences were statistically significant (HR=0.42; p = 0.0303) (Figure 3; Table 3B). The association between OS and FIGO stage, a classical OS prognostic factor, was not significant due to the small number of low-risk patients in this study (HR=2.92; p=0.074) (Table 3B). In multivariate analyses, a trend for a better survival was observed in patients with high tumor PTPN13 expression (HR=0.46; P=0.058) (Table 3B).

Finally, to strengthen these results, we assessed survival and tumor PTPN13 expression in a larger HGSOC dataset using the TCGA metadata on serous ovarian cancer samples (Grade 3) and the on-line Km-plotter tool [30]. This analysis showed a significantly longer survival for patients with high tumor PTPN13 expression (Supplementary Figure 2).

## DISCUSSION

HGSOC is the most common EOC subtype. Despite a high rate of clinical remission after cytoreductive surgery and chemotherapy, most patients will relapse and die of the disease [31]. The median PFS ranges from 17 to 30 months, and the median OS from 36 to 65 months, depending on the residual disease volume after surgery. Novel therapies are urgently needed to improve the outcome. Moreover, the identification of genes that are differentially expressed in ovarian carcinomas could yield potential biomarkers and also allow the identification of new therapeutic targets.

As the *PTPN13* gene is located on chromosome 4q that is frequently deleted in HGSOC [2, 5] and is a prognostic marker of survival in some cancers, we investigated PTPN13 expression profile in two cohorts of HGSOC samples by RT/PCR and by IHC, respectively. We found that in HGSOC, PTPN13 protein expression is heterogeneous and is reduced in one third of tumor samples compared with normal ovary epithelium.

In these two cohorts of patients with HGSOC and more than 8.5 years of follow-up, survival was significantly longer in patients with tumors showing high PTPN13 mRNA and protein expression. The HR values for OS in the two cohorts (0.27 and 0.42, respectively) were even more important than those obtained by a meta-analysis of the TCGA and HAS ovarian cohorts to identify genes that could be used as prognostic markers of ovarian cancer [32].

Only one previous study investigated PTPN13 expression in EOC [26] and did not find any significant prognostic interest of its expression, but the patients’ follow-up was limited to 2.3 or 5 years. Moreover, in this study, the prognostic effect of PTPN13 expression was not assessed in a specific EOC subtype, but in a heterogeneous cohort of EOC. This is important, because by analyzing *PTPN13* RNA expression in 12 endometrioid carcinoma samples, which have a better prognosis than HGSOC, we observed a heterogeneous expression with a slightly lower mean expression level than in HGSOC (data not shown). This illustrates the need for investigating the mechanisms underlying tumor development/progression in each specific EOC histopathological type.

Although based on two small retrospective cohorts and on the analysis of TCGA metadata from HGSOC samples, this study shows, for the first time, that high PTPN13 expression (mRNA and protein) in HGSOC is associated with favorable outcome. This finding and our previous mechanistic studies indicating that PTPN13 is an important regulatory element of human tumor growth and invasiveness suggest that PTPN13 suppression by 4q LOH enhances HGSOC aggressiveness. These results warrant further validation of PTPN13 prognostic importance in a larger prospective trial, and suggest a tumor suppressor role for PTPN13 in HGSOC. They also highlight the interest of studying its signaling in HGSOC to identify potential new therapeutic targets.

## MATERIALS AND METHODS

### Patients and tumor samples

Patients’ samples were selected from primary cytoreductive surgery specimens from two academic hospitals: the Santa Anna Hospital at the University of Torino for the RT-PCR study and the Institut regional du Cancer de Montpellier for the IHC study.

All patients were chemotherapy-naive at the time of surgery and were then treated with platinum-combined chemotherapy regimens as first-line treatment after surgery. At the time of relapse, standard chemotherapy regimens were used.

All patients were informed about the use of their tissue sample for research purpose and did not oppose. All samples were anonymized and expression analyses were performed blinded to the clinical and pathological data.

HGSOC and the tumor grade were defined according to the World Health Organization (WHO) criteria. The disease stage was evaluated according to the FIGO classification.

Cytoreductive surgery was defined as optimal in the case of residual disease ≤1cm and suboptimal in the case of greater residual disease.

### Cohort used for the RT-PCR analysis

Among the 63 patients with EOC treated at the University of Torino, between July 1991 and April 1999, 28 had a HGSOC. The patients’ median age at the time of surgery was 58 years (range, 25-71 years). Cytoreductive surgery was considered as optimal in 11 patients and suboptimal in 17 (Table 1A). Among the 20 patients with tumor relapse, 19 died and one was still alive at the end of the follow-up (median follow-up duration: 8.5 years). The eight patients without relapse were still alive at the end of the follow-up.

Fresh tumor samples were collected at the time of primary surgery. Specimens were snap-frozen in liquid nitrogen and stored at -80°C. Representative frozen sections from each tissue specimen were examined by two pathologists to confirm the tumor cell content. The tumor cells content ranged between 80% and 90%. The RNeasy Mini Kit (QIAGEN Inc., Valencia, Calif) was used to extract total RNA from 20 mg of tissue, manually pulverized in liquid nitrogen.

### Cohort used for IHC

Thirty HGSOC samples were selected from the ovarian carcinoma database of the Pathology Department of the Institut regional du Cancer de Montpellier. All samples were derived from archival blocks of paraffin-embedded tissue specimen. Twelve samples from non-tumor ovaries were selected from other patients and used as controls. The median age of the patients with HGSOC was 59 years (range 34–85), and 26 died during the study follow-up (median follow-up duration: 12.7 years) (Table 1B).

Tissue blocks with enough material upon gross inspection were initially selected and then the presence of carcinoma or normal ovarian surface epithelium was evaluated in hematoxylin–eosin-stained sections. The areas to be used for the construction of the Tissue Micro-Array (TMA) were marked on the slide and on the donor block. Samples corresponding to the selected areas were extracted using a manual arraying instrument (Manual Tissue Arrayer 1, Beecher Instruments, Sun Prairie, WI, USA). To take into account the tumor heterogeneity, tumor sampling consisted of three cores (0.6 mm in diameter) from different tumor areas from a single HGSOC specimen, at the specified coordinates. A single 2 mm tissue core of normal ovarian epithelium was sampled in a dedicated TMA. Finally, 4μm-thick sections were cut from the TMA blocks IHC.

### RT-PCR

RNA amount was quantified by measuring the absorbance at 260 nm. RNA quality was checked by assessing the ratio between the absorbance values at 260 nm and 280 nm, and was confirmed by RNA electrophoresis on 1.5% agarose gels containing ethidium bromide. Total RNA (2 μg) was reverse transcribed using 5 μM of random hexamers (Roche) and the Superscript II RNAse H reverse transcriptase, according to the manufacturer's instructions (Invitrogen). Real-time polymerase chain reaction (PCR) quantification of gene expression was carried out using cDNA corresponding to 12.5 ng of total RNA using a Light Cycler 3 device (Roche) with the Light Cycler FastStart DNA Master PLUS SYBR Green I Kit (Roche), according to the manufacturer's instructions. The primers used for PCR amplification were as follows: TATA Binding Protein (TBP) forward 5’ CAC GAA CCA CGG CAC TGA TT 3’(exon 4), reverse 5’ TTT TCT TGC TGC CAG TCT GGA C 3’(exon5) (product size 89bp, annealing temperature 61°C; median CTs= 27.91; PCR efficiency >98%); PTPN13 forward 5’ CAG TCA CAG AGA CCG AGC AGA CAA 3’ (exon 9-10), reverse 5’ TGC CGT TTT AGC ATG ATC TCT TGA 3’(exon 10) (product size 80bp, annealing temperature 61°C; median CTs = 27.85; PCR efficiency >98%).

The specificity of the chosen primer sequences was confirmed by using the BLASTN nucleotide-nucleotide tool and a database of expressed sequence tags and nr (the non-redundant set of the GenBank, EMBL and DDBJ database sequences). To check for amplification of contaminating genomic DNA, one of the two primers consisted of sequences derived from two adjacent exons. The relative quantification of PTPN13 gene expression was performed using the comparative cycle threshold (C_T_) method where the C_T_ parameter is defined as the number of cycles at which the fluorescent signal is first detectable. This method is based on the use of a calibrator sample and an endogenous RNA control, which allows the quantification of unknown samples. The human ovarian cancer cell line SKOV3, known to express PTPN13, was chosen as the calibrator sample (expression set at 1), and TBP mRNA was used as the endogenous RNA internal control. The relative PTPN13 expression was calculated using the 2^-ΔΔC^_T_ method [33], where ΔΔC_T_= ΔC_T_ patient sample - ΔC_T_ calibrator sample; with ΔC_T_= C_T_ PTPN13- C_T_TBP.

### IHC

Prior to immunostaining, antigen retrieval was performed by incubating the slides for 30min in EDTA pH9 antigen retrieval solution (Dako, Glostrup, Denmark) using a dedicated pre-treatment module (Dako). TMA immunostaining was then performed on a Techmate Horizon™ slide processor (Dako). After blocking endogenous peroxidase activity with Dako REAL™ Peroxidase-Blocking Solution, sections were incubated during 60 min at room temperature with antibody to PTPN13 (AC21 from AbCam 1/60). Immunostaining was revealed using the Envision DAKO® peroxidase/diaminobenzidine (DAB) visualization kit (30-min incubation). Slides were counterstained with hematoxylin (Dako). An immunoreactive score (IRS) was calculated based on the percentage of positive epithelial cells (<1%=0; 1-30%=1; 31-50%=2; 51–80%=3; >80%=4) multiplied by the staining intensity (negative=0; weak=1; moderate=2; strong=3), resulting in a value ranging between 0 and 12.

### Statistical analysis

Categorical variables were described using frequencies and percentages. Continuous variables were described using medians and range. Correlations between PTPN13 expression and clinical-pathological parameters were investigated using the chi-square test or Fisher's exact test.

OS was calculated from the date of surgery until the date of death or last contact. Patients alive or lost to follow-up were censored at the last contact date. PFS was calculated from the date of surgery to the date of detection of a new lesion by imaging, according to the Response Evaluation Criteria in Solid Tumors (RECIST), or the date of last contact in the case of non-relapse.

OS and PFS were estimated using the Kaplan-Meier method. Survival curves were compared using the log-rank test. Baseline prognostic factors for OS and PFS were evaluated in univariate analysis using a Cox proportional hazard model and were presented with HR and 95% CI. Significance was set at 0.05. All statistical analyses were performed using STATA version 13.0 (StataCorp).

## SUPPLEMENTARY MATERIALS FIGURES AND TABLES





## Abbreviations

EOC: epithelial ovarian carcinoma
HGSOC: high grade serous ovarian carcinoma
LOH: loss of heterozygosity
PTP: protein tyrosine phosphatase
WHO: World Health Organization
FIGO: International Federation of Gynecology and Obstetrics
PFS: progression-free survival
OS: overall survival
TMA: tissue micro array
PCR: polymerase chain reaction
CT: cycle threshold
TBP: TATA Binding Protein
IRS: immunoreactive score
RECIST: response evaluation criteria in solid tumors
IHC: immunohistochemistry
