# Supplementary material for: High PTPN13 expression in high grade serous ovarian carcinoma is associated with a better patient outcome
Source: Oncotarget. 2017 Sep 21;8(56):95662–73. doi: 10.18632/oncotarget.21175 (PMC5707051; doi:10.18632/oncotarget.21175)
Supplement: Supplementary file 1 [file oncotarget-08-95662-s001.pdf]

High PTPN13 expression in high grade serous ovarian carcinoma is associated with a better patient outcome

SUPPLEMENTARY MATERIALS

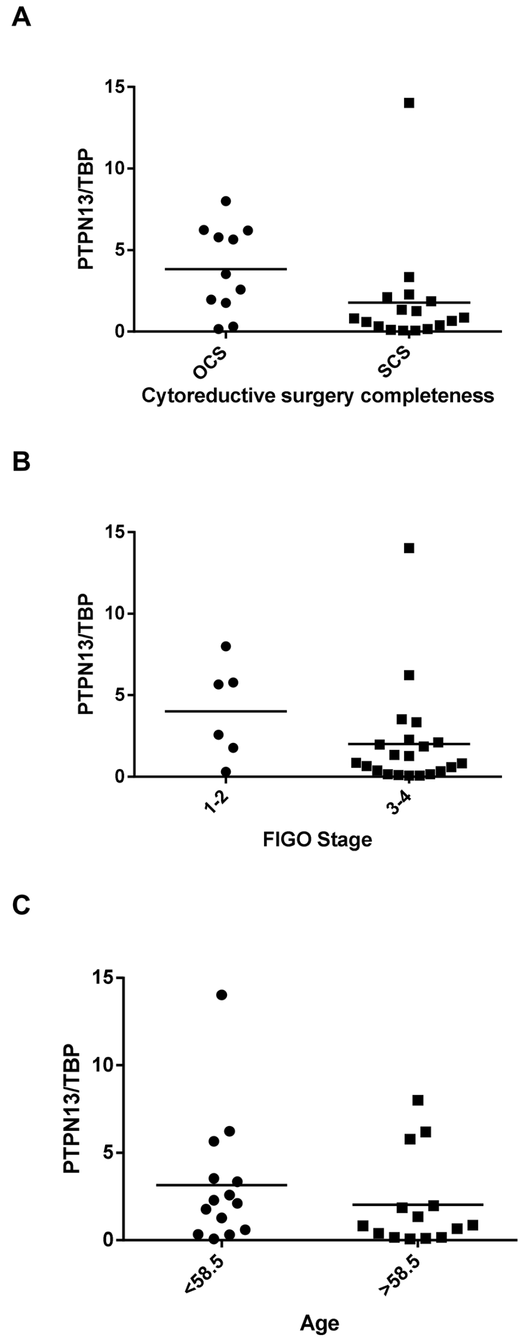

Supplementary Figure 1: Relative *PTPN13* mRNA expression in HGSOC samples. **(A)** According to cytoreductive surgery completeness. **(B)** According to the FIGO stage of the tumor. **(C)** According to the patients' age (see Table 1A).

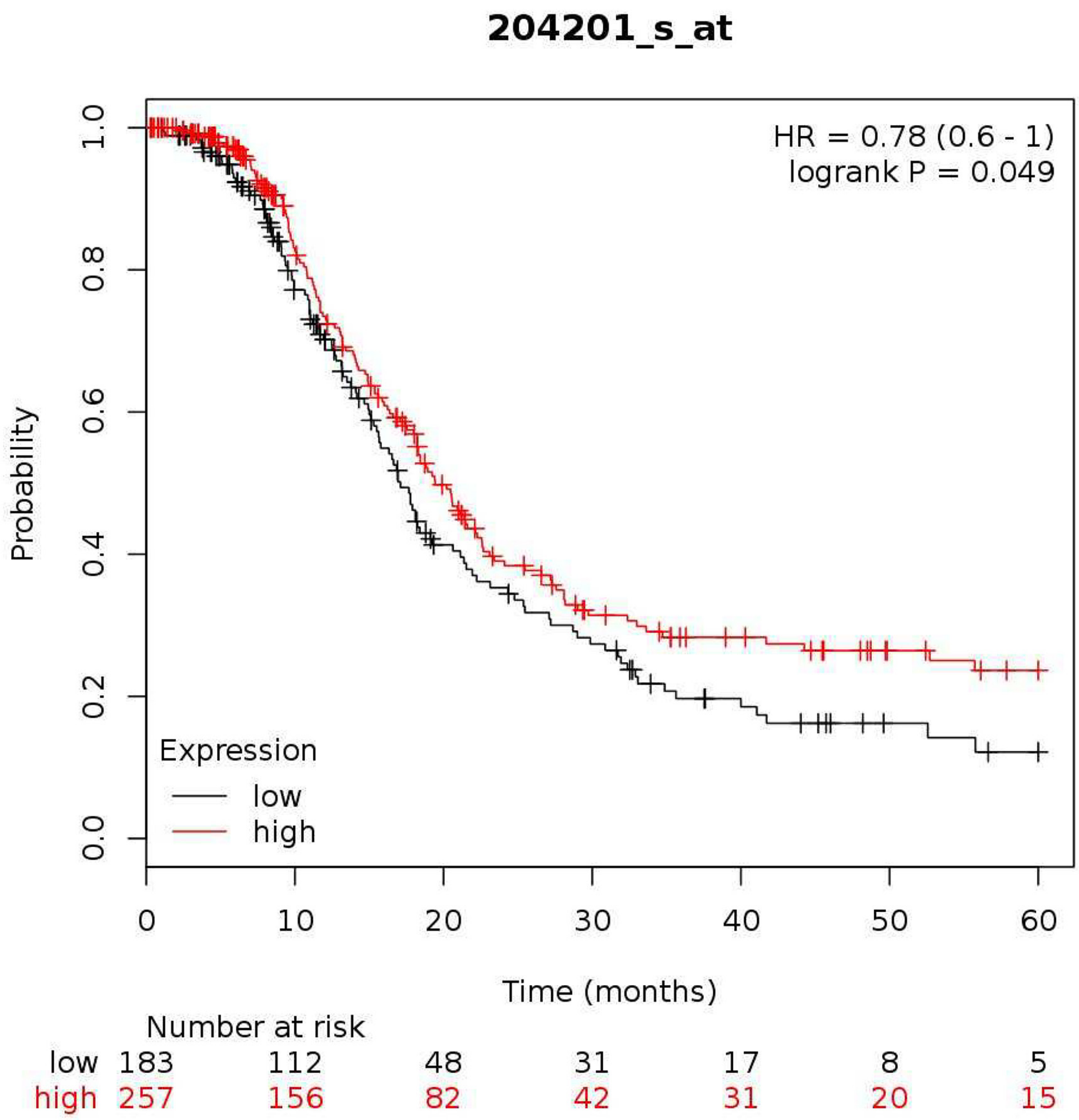

Supplementary Figure 2: Survival analysis was performed using TCGA metadata on ovarian cancer tumor samples (Serous, Grade 3) and the on-line Km-plotter tool. The Kaplan–Maier curves of progression-free survival time relative to the tumor PTPN13 expression (low/high), the hazard ratio with 95% confidence intervals and log-rank test P values at 5 years were calculated using the 204201_s_at probe set and automatically selected cut-offs.

Supplementary Table 1: Ct values obtained by quantitative RT-PCR. HGSOC samples are identified by a number. Standard: SKOV3; TBP: internal control.

See Supplementary File 1
